# Addressing the Adverse Effects of Climate Change on the Human Rights of Older Persons

**DOI:** 10.1093/geroni/igab046.2260

**Published:** 2021-12-17

**Authors:** Erin Burk-Leaver

**Affiliations:** International Association of Gerontology and Geriatrics, Denver, Colorado, United States

## Abstract

The common consequences of climate change events include: displacement, loss of sustainable shelter and housing, and limited access to medical care and other resources such as food, clean water, and sanitation services. These adverse effects coincide to an alarming degree with the human rights most essential to those in vulnerable or marginalized groups, including older populations. Whether through displacement or disruption of supply, the stressors of climate change events greatly exacerbate older populations’ vulnerability, especially when compounded by negative social determinants of health, such as existing social, political, and economic barriers to successful aging. Using the SDGs as a framework to develop policies around (13) climate action and the use of improved (9) industry, innovation, and infrastructure to create (11) sustainable cities and communities, it is possible to establish (10) reduced inequalities to promote overall (3) good health and well-being in our older populations.

